# Supplementary material for: Comparative genomics provides new insights into the diversity, physiology, and sexuality of the only industrially exploited tremellomycete: Phaffia rhodozyma
Source: BMC Genomics. 2016 Nov 9;17:901. doi: 10.1186/s12864-016-3244-7 (PMC5103461; doi:10.1186/s12864-016-3244-7)
Supplement: Additional file 6: — List of orphan genes with links to PFAM (related to Additional file 1: Table S1). (ZIP 1428 kb) [file 12864_2016_3244_MOESM6_ESM.zip › PFAM_UNIQUE/G04682_P.html]

#                                                                            --- full sequence --- -------------- this domain -------------   hmm coord   ali coord   env coord
  
# target name        accession   tlen query name           accession   qlen   E-value  score  bias   #  of  c-Evalue  i-Evalue  score  bias  from    to  from    to  from    to  acc description of target
  
#------------------- ---------- ----- -------------------- ---------- ----- --------- ------ ----- --- --- --------- --------- ------ ----- ----- ----- ----- ----- ----- ----- ---- ---------------------
  
G04682\_P             -            436 RHINO                PF15319.1    236       6.6    5.1  15.5   1   2     0.017        52    2.2   6.4    84   195   189   299   164   305 0.66 - RAD9, RAD1, HUS1-interacting nuclear orphan protein  
G04682\_P             -            436 RHINO                PF15319.1    236       6.6    5.1  15.5   2   2   0.00025      0.75    8.2   1.4    95   157   337   402   326   432 0.79 - RAD9, RAD1, HUS1-interacting nuclear orphan protein
